# Pathogenesis, Diagnosis, and Treatment of Infectious Rhinosinusitis

**DOI:** 10.3390/microorganisms12081690

**Published:** 2024-08-16

**Authors:** Fujiao Huang, Fangyan Liu, Xiaofang Zhen, Shu Gong, Wenbi Chen, Zhangyong Song

**Affiliations:** 1 School of Basic Medical Sciences, Southwest Medical University, Luzhou 646000, China; 2 The Public Platform of Cell Biotechnology, Public Center of Experimental Technology, Southwest Medical University, Luzhou 646000, China; 3 Molecular Biotechnology Platform, Public Center of Experimental Technology, Southwest Medical University, Luzhou 646000, China; 4 Hemodynamics and Medical Engineering Combination Key Laboratory of Luzhou, Luzhou 646000, China

**Keywords:** fungi, bacteria, virus, rhinosinusitis, immunology

## Abstract

Rhinosinusitis is a common inflammatory disease of the sinonasal mucosa and paranasal sinuses. The pathogenesis of rhinosinusitis involves a variety of factors, including genetics, nasal microbiota status, infection, and environmental influences. Pathogenic microorganisms, including viruses, bacteria, and fungi, have been proven to target the cilia and/or epithelial cells of ciliated airways, which results in the impairment of mucociliary clearance, leading to epithelial cell apoptosis and the loss of epithelial barrier integrity and immune dysregulation, thereby facilitating infection. However, the mechanisms employed by pathogenic microorganisms in rhinosinusitis remain unclear. Therefore, this review describes the types of common pathogenic microorganisms that cause rhinosinusitis, including human rhinovirus, respiratory syncytial virus, *Staphylococcus aureus*, *Pseudomonas aeruginosa*, *Aspergillus species*, etc. The damage of mucosal cilium clearance and epithelial barrier caused by surface proteins or secreted virulence factors are summarized in detail. In addition, the specific inflammatory response, mainly Type 1 immune responses (Th1) and Type 2 immune responses (Th2), induced by the entry of pathogens into the body is discussed. The conventional treatment of infectious sinusitis and emerging treatment methods including nanotechnology are also discussed in order to improve the current understanding of the types of microorganisms that cause rhinosinusitis and to help effectively select surgical and/or therapeutic interventions for precise and personalized treatment.

## 1. Introduction

**Figure 1:**
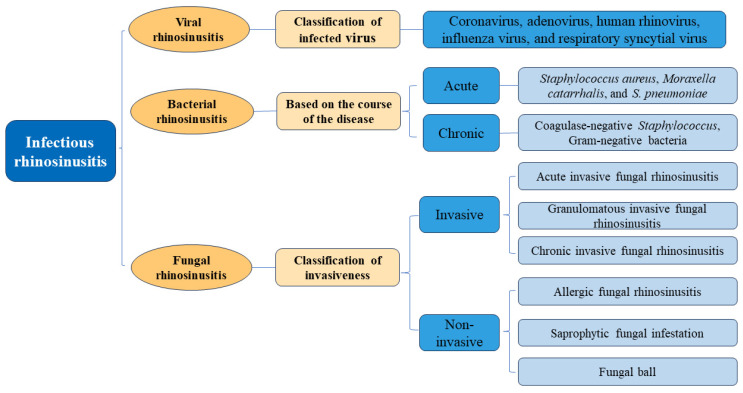
Classification of infectious rhinosinusitis.

Rhinosinusitis (RS) is an inflammation of the mucosa of the nose and paranasal sinuses. RS affects about 15% of the population annually worldwide and has a high incidence and disease burden, which adversely affects quality of life [1,2,3]. The onset and development of RS are facilitated by infection as well as genetic and environmental factors. Based on duration, there are three types of RS: acute, subacute, and chronic (ARS, SRS, and CRS, respectively) [4]. ARS refers to any short-term (<4 weeks) persistent inflammation of the lining of the nose and sinuses. The most common cause of ARS is infection, primarily from viruses, but it can also be caused by bacteria and fungi. The duration of SRS is typically 4 to 12 weeks but sometimes more [1,4]. ARS was responsible for about 21.4 million outpatient visits from 2006 to 2010, while about 47.9 million visits were associated with a primary diagnosis of CRS, of which antibiotics were administered in 85.5% and 69.3% of ARS and CRS patients, respectively [5]. Another survey revealed that more than 80% and 50% of patients with ARS and CRS, respectively, were treated with antibiotics [6,7]. Clinical suspicion of infection is the foundation for the diagnosis of CRS. In this review, infectious RS is specifically defined as the acute exacerbation of RS caused by identifiable infectious pathogens. Figure 1 illustrates the classification of infectious RS. Although the mechanisms by which pathogens cause RS are not completely understood, this review aims to elucidate the roles of viruses, bacteria, and fungi in RS pathogenesis and clarify the clinical characteristics, host immune responses, diagnosis, and treatment strategies to provide alternative viewpoints for the clinical diagnosis and treatment of infectious RS. As far as we know, this review is the first to focus systematically on the various pathogens that cause RS, summarize the diagnosis of different types of infections with RS, and discuss emerging treatments.

## 2. Viral RS

Upper respiratory tract infections most often result from viruses and most cases resolve spontaneously. However, CRS is usually caused by upper respiratory tract infection, with progression of symptom severity and duration. Respiratory viruses are detected in mucosa or lavage fluid of about 50–70% of CRS patients [8,9]. Moreover, viral infection causes obstruction of the sinus opening, production of inflammatory mediators by nasal epithelial cells (ECs), damage to ECs and cilia, sustained changes to local cytokine production, and increased bacterial adhesion to nasal ECs [10]. A virus binds to the sinonasal epithelium through specific receptors, then enters the cell and replicates. Viral infection may be related to the sustained high reactivity of sinus mucosa and susceptibility to bacterial infections. In addition, viral infection triggers an immune response and upregulation of genes associated with airway remodeling, thereby exacerbating CRS [11]. Chronic inflammation, a decrease in mucociliary clearance, and a loss of epithelial barrier (EB) function are characteristics of the host immunological response to viral infection [12].

Coronavirus, adenovirus, human rhinovirus (HRV), influenza virus, and respiratory syncytial virus (RSV) are the most common causes of RS. HRV typically infects the upper respiratory tract. There are more than 160 HRV serotypes, which are classified into three groups: HRV-A, HRV-B, and HRV-C [13]. Interactions between the host genetics and HRV could have a significant impact on how severe RS is. Various clinical strains of HRV-A, HRV-B, and HRV-C are known to attack ECs at the gas–liquid interface of the paranasal sinuses. HRV-B strains replicate at significantly lower rates, promote a milder host immune response, and are less cytotoxic to cells than HRV-A and HRV-C, supporting clinical observations that HRV-A and HRV-C strains cause more severe disease than HRV-B strains [14]. The second most frequent virus that causes sinus infections is RSV. In addition, although RSV infection is not age-specific, airway morbidity and mortality are significantly higher in infants, children, and the elderly due to damaged antiviral host responses [12].

These viruses attach themselves to nasal airway receptors that mediate cell entry, which triggers host immune responses. The presence of viral infection can lead to an inflammatory reaction, which can be identified by symptoms such as fever, nasal congestion, reduced sense of smell, face discomfort and/or pressure, and the discharge of mucus from the back of the nose. Respiratory viruses are commonly detected in CRS individuals, with rhinoviruses accounting for about 60–70% of CRS cases (11). However, viral upper respiratory tract infections can progress to bacterial RS and exacerbate CRS [8,9,15]. In addition, HRV infection can disrupt the microbial composition of the airways. For example, HRV infection leads to increased proportions of *Dolosigranulum* and *Moraxella* [16]. Meanwhile, rhinovirus infection can significantly alter the expression of C-C and C-X-C family genes, which control the recruitment of inflammatory cells.

### 2.1. The Pathogenesis of Viral RS

Viruses can play three distinct roles in the development of inflammation in CRS: initiating the inflammation, continuously stimulating the inflammation, and causing the sudden worsening of symptoms [10]. However, the specific roles of various viruses in RS remain unclear.

#### 2.1.1. Virus-Specific Binding to Host Receptors

Respiratory viruses are transmitted through the mucosal surface of the nose or mouth via binding of the fusion protein on the viral envelope to nasal ECs through specific cell surface receptors. HRVs invade host cells through three cell membrane glycoproteins: intercellular adhesion molecule-1 (ICAM-1), low-density lipoprotein receptor (LDLR), and cadherin-related family member 3 (CDHR3) [17]. ICAM-1 is an immunoglobulin superfamily member that is expressed by endothelial cells, leukocytes, and ECs. It facilitates leukocyte adherence to endothelial cells as well as EC migration, barrier function, and proliferation [18,19]. The LDLR family members are a set of cell surface receptors involved in endocytosis that specifically bind to ligands found outside the cell. Cadherins are a cluster of glycoproteins that are embedded in the cell membrane and have a role in cell adhesion, cell signaling, and mechanical transduction. CDHR3 receptors exhibit a significant level of expression on the plasma membrane of airway epithelial cells [20]. A single nucleotide polymorphism (rs6967330) of *CDHR3* was reported to promote HRV-C infection and the occurrence of infantile diseases, thereby affecting the developing lung and increasing the risk of asthma. The rs6967330 allele was linked to increased HRV-C binding, replication, and total protein expression [17,21]. Moreover, the rs6967330 single nucleotide polymorphism is associated with a two-fold increased risk of CRS [22].

RSV enters cells by attaching to the CX3CR1 receptor on the heparin sulfate proteoglycan of airway cells through an attachment glycoprotein (G protein) [23]. The RSV F protein adheres to nucleolin, insulin-like growth factor-1 receptor, epidermal growth factor, and ICAM-1 before entering the cell [24]. The hemagglutinin (HA) protein of influenza viruses binds to sialic acid-containing receptors on various cell types, including the airways. In addition, influenza viruses cause red blood cell agglutination through hemagglutinin, a viral attachment protein. Influenza viruses also produce the surface protein neuraminidase, which cleaves sialic acid after binding to any molecule that does not cause viral infection, thereby releasing the virus. Influenza viruses target human airway ECs primarily through α-2,6 receptors [25]. Moreover, different coronaviruses bind to epithelial cells by different receptors. Human coronavirus HCoV-229E invades cells through human aminopeptidase N [26]. Angiotensin-converting enzyme 2 (ACE2) is used by the severe acute respiratory syndrome coronavirus (SARS-CoV) and SARS-CoV-2 to enter cells [27]. The high expression and broad distribution of TMPRSS2 in human organs help to active the SARS-CoV-2 spike protein and facilitate cellular entry and virus-cell fusion [28,29]. Angiotensin (Ang) II is catalyzed and inactivated by ACE2, and tissue damage is encouraged by Ang II through inducing vasoconstriction, cytokine production, and apoptosis [30]. Nasal epithelial cells exhibit a significant expression of ACE2 [27]. HRV infection increases the expression of ACE2 in individuals with asthma [31], suggesting that HRVs, RSVs, and influenza viruses may also increase the expression of ACE2 and the harshness of coronavirus disease 2019 (COVID-19). However, further evidence is required to confirm this hypothesis. Investigations have revealed that individuals with CRS have a higher chance of contracting SARS-CoV-2 and developing severe COVID-19 [32]. ACE2 expression was elevated in nasal tissue of non-eosinophilic CRS patients [33,34].

#### 2.1.2. Impairment of Ciliary Clearance and EB Destruction

Mucosal cilia clearance and EB function play important roles in resisting viral invasion. Mucus produced by goblet cells and submucosal glands clear the trichoid motile cilia lining the airways [35]. SARS-CoV-2 infection results in a transient decrease in EB function, the disruption of tight junctions (TJs), and the impairment of motile ciliary function [36]. Infection with respiratory viruses downregulates the expression of key genes involved in intrafilial transport and ciliary dynein machinery [37]. SARS-CoV and SARS-CoV-2 can destroy motor cilia by downregulating *FOXJ1*, a transcription factor necessary for cilia [29]. Prior investigations using experimental models have confirmed that influenza virus infection leads to the apoptosis of ECs, loss of ciliated cells, and thinning of cell thickness [38]. In addition, RSV can cause increased ciliary dyskinesia, accompanied by cilia loss and epithelial damage, thus leading to a substantial decrease in mucociliary clearance [39]. RSV viral surface glycoproteins can cause mucus production to rise and mucociliary clearance to slow down because they bind to the CX3CR1 receptor found on cell motile cilia and enhance the expression of mucins, such as MUC5AC and MUC5B [40]. It is very important that respiratory viruses and cilia-related processes interact. Why a prior viral infection makes the host more vulnerable to secondary coinfection with another pathogen is explained by the impairment of EC mucociliary clearance.

Zonula occludens-1 (ZO-1), occludin, and other TJ proteins that mediate paracellular transport and adherens junction formation make up the integral barrier of the airway epithelium. By attaching to the actin cytoskeleton, these proteins enable cellular integrity and intercellular adhesion. HRV infection leads to a significant reduction in mRNA levels of the components of TJs and adhesive connections [41]. RSV infection leads to the enhanced permeability of the airway epithelium and destruction of TJ structures, leading to airway barrier dysfunction [42]. Moreover, influenza viruses have been demonstrated to compromise the integrity of the airway epithelium and disrupt the TJ protein ZO-1’s connection between the actin cytoskeleton and the transmembrane protein occludin [43]. Prior investigations have confirmed that viral infections and/or colds, allergies, and asthma are substantial risk factors for early onset CRS and that destruction of the EB plays a key role in the pathophysiology of CRS [44,45,46].

#### 2.1.3. Host Immune Imbalance

Viruses activate the host immune response via various mechanisms. The infection of ECs by upper respiratory viruses activate immune cells to release cytokines, which cause inflammatory changes to the infected area. In addition, viral infection and lymphocyte clearance can exacerbate epithelial damage [47]. The host cell’s pattern recognition receptors are activated to start the innate immune response. Viral RNA can activate about 40 cytoplasmic receptors and Toll-like receptors (TLRs) on the cell surface. Following the activation of signal integrators by these pattern recognition receptors, the production of genes encoding for inflammatory cytokines and antiviral IFNs is encouraged [48,49]. All three types of IFNs can directly or indirectly mediate antiviral responses [50,51]. While IFN production is essential for innate immunity against viruses, there is evidence that it may also be important in chronic inflammation [51]. HRV infection of human nasal ECs induces the expression of CXC motif chemokine ligand (CXCL)-11, inducible protein-10, CXCL-9, and RANTES, and activates TLR7 and RIG-I [52,53]. However, an in vitro investigation found that no difference in the generation of antiviral IFNs induced by HRV between the healthy group and CRS groups [54]. In addition, in CRS individuals, the expression levels of type I and III IFNs and IFN-stimulated genes are decreased, suggesting that IFN expression levels are inconsistent [55]. According to a study on asthma, those with asthma had lower baseline levels of IFN production, which led to increased viral replication and an inflated IFN response during an asthma attack [56].

Th2 are characterized by the recruitment and activation of mast cells, basophils, and eosinophils and increased production of immunoglobulin E (IgE) and ILs. A previous study showed that HRV infection prompted the production of IL-33 and subsequent secretion of the type 2 cytokines [57]. Likewise, it has been shown that RSV infection increases the levels of IL-4, IL-6, and IL-13 in children’s nasal rinse [58]. In addition, it has been demonstrated that RSV infection raises the levels of IL-33 and IL-13 in hospitalized newborns as well as IL-33 type 2 intrinsic cells in newborn mice [59].

In summary, the disruption of the EB and immune responses renders the nasal mucosa more vulnerable to antigen exposure and activation, while increasing susceptibility of the epithelium to viruses, thereby promoting disease progression and increasing the likelihood of bacterial infection.

### 2.2. The Diagnosis and Treatment of Viral RS

**Figure 2:**
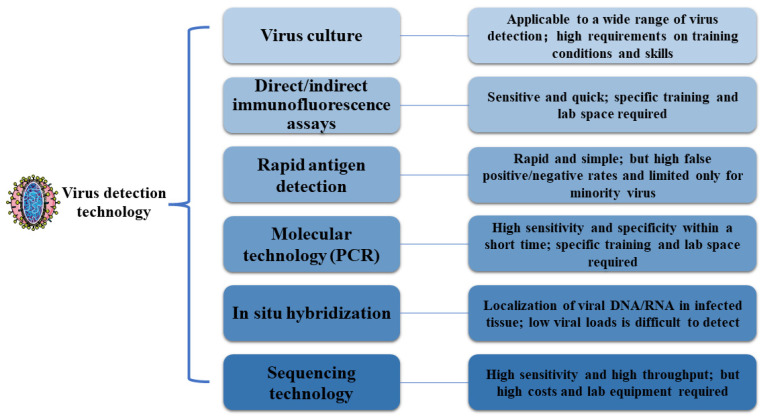
Comparison of several different detection techniques for viruses.

Since symptoms are often non-specific, viral RS is mainly diagnosed based on clinical manifestations of sinus inflammation, sinus computed tomography (CT), and molecular detection technology. Methods used for the detection of viruses mainly include traditional virus culture and direct/indirect immunofluorescence assays, rapid antigen detection, and highly sensitive nucleic acid amplification [60]. Rapid virus detection culture facilitates the detection of adenoviruses, influenza viruses, and RSV within 48 h [61]. The rapid direct antigen test is simple to perform but limited for the detection of influenza viruses and RSV [62,63]. The viral detection techniques with their advantages and disadvantages are shown in Figure 2. Since most cases of RS are acute, treatment is usually limited to antiviral drugs and surgery. In addition to the usual use of antiviral drugs, there are some emerging therapies. Ozone has been reported to inactivate both herpes simplex virus type 1 and the hepatitis C virus, and combined treatment with ozone and antiviral drugs has shown a reduction in inflammation and lung damage [64,65]. Antiviral antibodies made in a recovering patient’s plasma are injected into the patient as part of convalescent plasma treatment. The treatment has shown efficacy in H5N1 influenza and Ebola virus disease, as well as improved cases of severe COVID-19 [66,67,68,69]. Research has confirmed a greater advantage for nanotechnology-based drug delivery in treating mice infected with SARS-CoV [70,71].

## 3. Bacterial RS

Bacterial RS is an infection of the nasal epithelium and paranasal sinus mucosa, usually caused by *Streptococcus pneumoniae* and *Haemophilus influenzae.* Bacterial RS can be categorized as acute or chronic based on how the illness progresses. The differences between acute and chronic bacterial RS mainly include the following points: the duration of acute bacterial RS is generally not more than 12 weeks, while the duration of chronic bacterial RS is longer, mostly more than 3 months. Generally, chronic bacterial RS is more common than acute bacterial RS. The most common bacteria are *Staphylococcus aureus*, *Moraxella catarrhalis*, and *S. pneumoniae* in acute RS. Coagulase-negative *Staphylococcus*, Gram-negative bacteria, and anaerobic bacteria are the major causes of chronic bacterial RS. *Pseudomonas aeruginosa* is reportedly more common in those who have a history of sinus surgeries [72]. Moreover, sinonasal colonization by these bacteria can exacerbate inflammation.

### 3.1. The Pathogenesis of Bacterial RS

#### 3.1.1. Bacterial Invasion of the Epithelium

In the initial phases of infection, bacteria disrupt mucosal barrier function by decreasing ciliary irritability and the frequency of basal cilia beats. Various virulence factors generated by *S. aureus* can effectively evade the host immune response while activating non-specific inflammatory responses. In addition, bacterial exo-proteins have been shown to disrupt EB function via downregulation of genes encoding transmembrane proteins [73]. Bacterial exo-proteins also disrupt the function of TJ proteins, resulting in the increased permeability of polarized ECs and could also directly or indirectly induce proinflammatory cytokines to disrupt the structural integrity of TJs and the EB. For example, *S. aureus* releases various exotoxins, including toxic shock syndrome toxin 1, *Staphylococcus* enterotoxins, and *Staphylococcus* superantigen-like toxins [74]. Moreover, staphylococcal enterotoxin B (SEB) exhibits superantigen properties that directly affect immune stimulation via the recruitment of effector cells and the generation of inflammatory cytokines. In addition, SEB upregulates the production of IL-6 by activating TLR2 [73]. As a possible mechanism of SEB, mast cells ingest *S. aureus* for degradation. However, non-degraded but active *S. aureus* combined with SEB stimulates the degranulation of mast cells and release pro-inflammatory mediators and cytokines [75]. Moreover, SEB induces the production of reactive oxygen species (ROS) in the ECs of CRS patients. The exposure of nasal ECs to SEB increases the production of mitochondrial ROS. In fact, inflammation is associated with the generation of ROS, which is related to the induction of endoplasmic reticulum stress and the development of nasal polyps [76,77,78]. Similarly, elastase and serine proteases released by *P. aeruginosa* has been correlated with increased permeability, decreased transepithelial electrical resistance, and the severity of CRS [79,80,81]. The transmembrane proteins tricellulin, occludin, and claudin-1 and -4 were reduced by the elastase, which also damaged the epithelial barrier. Additionally, *P. aeruginosa* elastase decreased PAR-2 expression, which in turn controlled the production of TJ proteins [82]. Bacterial biofilms can significantly damage the sinonasal cilia and are found in the sinuses of about 42–80% of CRS patients [83]. Moreover, biofilm formation plays a role in the severity and persistence of CRS and the failure of antibiotic therapy [84]. Oral antibiotics are difficult to use against deep bacterial biofilms, and systemic high concentrations of drugs may be toxic and also affect commensal microorganisms and cause resistance to antibiotics [74,85].

In addition, the breakdown of the epithelial barrier and dysregulation of mucin expression are hypotheses of biofilm involvement in CRS formation [86]. When the epithelium barrier is destroyed, the body is exposed to more microbes, and inflammatory reactions are overstimulated. Multiple mucins in CRS are dysregulated in their expression, and these mucins serve as bacterial adhesin attachment ligands, triggering bacterial colonization [87]. The incidence of bacterial biofilm in CRS is significantly higher. Moreover, the expression level of MUC5B is increased, indicating a certain relationship between biofilm and mucin expression [88]. The most often detected bacteria that form biofilms in CRS individuals include *S. aureus*, *S. pneumoniae*, *H. influenzae*, and *P. aeruginosa*. Bacterial biofilms are also detected in healthy groups, suggesting that biofilms could be a normal respiratory tract element [89]. However, other investigations have suggested that patients with CRS who develop biofilms may have a poor prognosis and disease resistance. For example, the quality of life of CRS individuals with bacterial infection and biofilm formation after functional endoscopic sinus surgery (FESS) was significantly improved at the initial stage, although there was no noticeable distinction in CRS patients without biofilm formation at 6 months after FESS [74,90]. Refractory CRS is defined by the increased recruitment of CD4^+^ helper T cells and biofilm production by *S. aureus* [91]. In addition, the extracellular proteins of the *S. aureus* biofilm decrease transepithelial electrical resistance, increase cytotoxicity and permeability, and disrupt TJ proteins in a time- and dose- dependent manner. These alterations could lead to mucosal inflammation in CRS patients [92]. Moreover, biofilm produced by *S. aureus* is linked to eosinophil markers of inflammation, such as eosinophil cationic protein and IL-5, indicating the possible significance of staphylococcal biofilms in TH2 inflammation in chronic rhinosinusitis with nasal polyps (CRSwNP) [93].

#### 3.1.2. Host Immune Response

The disruption of the nasal EB by virulence factors and other components of bacteria triggers an inflammatory response by the host cell. Bacterial virulence factors function as superantigens and can bypass processing by antigen-presenting cells and attach directly to major histocompatibility complex class II molecules and T cell receptors, thereby activating many T cells, accompanied by B cell proliferation and eosinophil activation [94]. The activation of B cells upregulates the expression of Th2 cytokine, causing the synthesis of polyclonal IgE and the release of histamine. Clinically, significant elevations of the key proinflammatory cytokines TNF-α, IFN-γ, and IL-13 were detected in inferior turbinate tissue supernatants of CRS patients with SEB, indicating the presence of a type 2 inflammatory phenotype [94,95]. In summary, bacteria increase the severity of RS mainly through biofilm formation and also regulate immune responses via the disruption of tissue barrier function and the impairment of mucociliary clearance, which promotes the development of polyps and type 2 inflammation [96].

### 3.2. The Diagnosis and Treatment of Bacterial RS

The main methods used to diagnose bacterial RS are sinus CT, clinical symptoms, and microbial culture. Viral and bacterial ARS are often followed by many common cold symptoms, such as sore throat, cough, fever, and sinus irritation. Furthermore, the symptoms of bacterial ARS are generally worse than a common cold and may cause severe one-sided facial pain, high fever (>38°), and greenish yellow to rust brown mucus. Viral RS is generally acute, while the symptoms of bacterial RS are often longer lasting. The symptoms of severe or long-lasting CRS caused by an acute bacterial infection include nasal blockage/congestion, nasal discharge, altered sense of smell, and facial pain. For patients without sinus disease, the sinus spaces are full of air and will appear black on a CT scan. In CRSwNP individuals, on a CT scan, the sinus lining appears grey instead of black due to edema and inflammation, and the sinuses may appear partially or entirely filled with polyps or mucus [1].

Treatment options include topical intranasal steroids, oral antibiotics, topical antibiotics, nasal saline irrigation, oral steroids, or combinations of oral antibiotics and steroids. The matrix components of bacterial biofilms protect the bacteria from antibodies, phagocytosis, and antibiotic killing [97,98]. According to reports, individuals with CRS biofilm responded less well to high antibiotic dosages and experienced more FESS revisions [85,99]. In recent years, natural products have the advantage of being less toxic, and some researchers have reported the potential role of natural products in inhibiting bacterial biofilms; for example, honey and and active component of honey (methylglyoxal) significantly decreased *S. aureus* biofilm in a sheep sinusitis model [100]. Other natural products, including 1,8-cineole and xylitol, have also been used to prevent the development of regular sinonasal bacterial biofilms [101,102,103]. A higher concentration of NO can reduce the biomass of *S. aureus* biofilm isolated from CRS individuals [104,105]. In addition, it has been reported that phage therapy can overcome antibiotic resistance in chronic rhinosinusitis [106]. Although these new treatments have some effect on the clearance of CRS bacteria, additional clinical research is required to justify their wider use. Surgery is required for refractory cases, and immunoglobulin replacement therapy may be required for those with underlying immunodeficiency [107,108].

## 4. Fungal RS

Fungal RS is a general term describing fungal diseases of the nasal cavity and sinuses. The main predisposing factors of fungal RS are antibiotic use, corticosteroid treatment, and diabetes. In contrast to bacterial and viral RS, the most typical signs and symptoms of fungal RS include ocular swelling, exophthalmos, and headache [109]. Depending on the presence of pathogenic fungi, fungal RS is classed as invasive or non-invasive. Invasive fungal RS includes acute invasive fungal rhinosinusitis (AIFRS), granulomatous invasive fungal rhinosinusitis (GIFRS), and chronic invasive fungal rhinosinusitis (CIFRS). The symptoms of invasive fungal RS include fever, coughing, and sometimes nasal mucosal ulcers [110,111]. Histopathological studies show that fungal RS is characterized by hyphal invasion of the sinus mucosa, submucosa, and blood vessels with vasculitis, thrombosis, and tissue infarction [112,113,114]. This aggressive form is often seen in immunocompromised individuals, older people, and those with intracranial involvement, who usually have a poor survival rate. Non-invasive fungal RS includes allergic fungal rhinosinusitis (AFRS), saprophytic fungal infestation, and aspergilloma (fungal ball) [111,115]. Non-invasive fungal RS presents as CRS that does not respond to repeated antibiotics and surgery [116]. A retrospective study of 400 patients with fungal RS reported that 87.5% were non-invasive lesions, of which 40% were fungal spheroid, 45% were AFRS, 12.5% were invasive RS, and 11.0% were AIFRS. Moreover, *Aspergillus* was the most common fungus isolated from aspergilloma, AFRS, and AIFRS patients [117,118].

### 4.1. Invasive Fungal RS

#### 4.1.1. AIFRS

AIFRS is a rare but deadly illness brought on by an invasive fungal invasion of the paranasal sinuses and nasal cavity. Fungal hyphae appear in the mucosa, submucosa, blood vessels, or bones of the sinus and subsequently spread into the orbit and brain within a few hours [111,114,119]. The survival rate of AIFRS is only 20–80% [119,120,121]. Pathogens causing AIFRS include typical *Mucor*, *Aspergillus*, and atypical fungi (*Alternaria*, *Candida*, *Fusarium*, *Paecilomyces*, *Scedosporium*, and *Scopularopsis*) [122,123,124]. Previous investigations confirmed that susceptibility to AIFRS is increased in patients with host cell-mediated immune deficiency, especially neutropenia, hematological malignancies, aplastic anemia, hemochromatosis, poorly controlled diabetes, liver and kidney failure, acquired immunodeficiency syndrome, and organ transplantation, as well as those receiving immunosuppressive therapy with systemic steroids or chemotherapeutic agents [114,125,126]. An analysis of 807 individuals discovered that the most common symptoms of AIFRS include facial swelling, fever, nasal congestion, and orbital lesions in about half of patients, altered mental status and palate necrosis in a small percentage of patients, and enucleation of the eye in about 19.8%. Although antifungal medication and early surgical debridement are used in the treatment of most AIFRS patients, the overall survival rate is only 49.7%. However, individuals who received liposomal amphotericin B or had open or endoscopic sinus surgery had a higher survival rate. Reported poor prognostic factors include intracranial involvement, treatment delay, altered mental status, eye socket resection, advanced age, diabetic ketoacidosis, and drug resistance [116,127]. Therefore, early diagnosis and treatment are crucial to decrease the morbidity and mortality associated with AIFRS.

##### Pathogenesis and Host Immunity

Anatomical abnormalities, environmental exposures, microbial toxicity, genetic background, and immune state have all been suggested as underlying causes of AIFRS, even though the pathogenesis and etiology of the condition are still unknown. The type of nasal mycosis is primarily determined by immunological condition. Crucially, it is still unknown what pathophysiological pathways lead from harmless fungal colonization to fungal RS.

##### Diagnosis of AIFRS

Clinical symptoms: Common symptoms of AIFRS include acute nasal obstruction for a month or less, facial swelling, and fever. Other less common symptoms include facial pain/numbness, headache, eye paralysis and protrusion, changes to vision, and significant mental changes [122,128]. Maxillary involvement has been found to be significantly linked with mortality [129]. However, these symptoms are not specific and often overlap with other diseases and bacterial/viral RS.

Microbiological examination: Fungal species are considered important prognostic factors, with *Aspergillus* and *Mucor* as the most common causes of AIFRS [130]. Previous investigations reported that *Mucor* infection is predictive of worse outcomes. As compared to infections by *Aspergillus* and *Fusarium*, individuals with diabetes are more likely to have an infection with *Mucorales*. In addition, baseline creatinine levels are greater in patients with invasive fungal RS caused by *Mucorales* infection than in patients with *Aspergillus* and *Fusarium* infections [131]. Atypical fungal infections have been associated with a worse 1-month survival [124]. However, microbial culture is time-consuming, and only 36% of confirmed cases have positive culture results [132]. In addition, detecting fungal infections can be done using molecular biology methods, including real-time fluorescence quantitative polymerase chain reaction (PCR) for *Aspergillus* and *Candida*, and nested PCR for *Mucorales* [133]. A previous investigation reported that the culture technique alone detected fungal components in 25% of nasal lavage fluid samples, while the PCR technique alone found fungal DNA in 44% of nasal lavage fluid samples, whereas the positivity rate was improved to 50% by the combination of the culture and PCR techniques. Although PCR analysis significantly improved the fungal detection rate of nasal lavage fluid samples, the culture technique remains the gold standard [134,135]. In addition, the identification of blood-based fungal indicators such as galactomannan or β-glucan is important for diagnosing invasive aspergillosis; nevertheless, its specificity and sensitivity are restricted. Moreover, there was no noticeable difference in peak galactomannan levels of patients with positive versus negative pathological results for AIFS. False-positive results of elevated galactomannan can occur due to various factors, such as diet, other fungi, and the use of certain antibiotics. Therefore, caution is recommended with the use of galactomannan to diagnose AIFRS [136].

Laboratory tests: Absolute neutropenia and diabetes mellitus are primary risk aspects for AIFRS. Therefore, dynamic monitoring of basal metabolism markers and assessment of blood glucose/ketoacidosis is important for diagnosing RS. Unexpectedly, increased glycated hemoglobin (HbA1c) was linked to better survival, possible because higher HbA1c is a more treatable and reversible cause of immunosuppression. However, further investigations are needed for confirmation [124]. Meanwhile, blood analysis is also required to assess the absolute neutrophil count (ANC). In a previous study, 82.9% of 41 patients with AIFRS had an ANC < 500/μL [128]. Although investigations have found no significant relationship of ANC < 500 μL with the diagnosis and prognosis of AIFRS, ANC < 500 μL remains an important risk factor for AIFRS [124]. Moreover, C-reactive protein levels are significantly associated with the survival of patients with AIFRS [129,137,138]. Therefore, it is important to closely monitor the indicators of acute inflammation.

Imaging and nasal endoscopy: CT and magnetic resonance imaging (MRI) are commonly used for the diagnosis of the sinuses. Through CT, AIFRS is characterized by the severe edema of the mucosa and soft tissues and thickening of the sinus mucosa. The most reliable CT result is unilateral nasal mucosal thickening, and these occur more frequently in immunocompromised than immunocompetent patients [139]. MRI is a more sensitive diagnostic method for AIFRS than CT. As an inherent advantage, the resolution of soft tissue contrast is better with MRI than CT. Therefore, MRI can more accurately distinguish mucus from edema when evaluating the intracranial and orbital infiltration of AIFRS. Signs of AIFRS described by MRI include inflammation of the orbital fat and extraocular muscles with leptomeningeal enhancement [140]. The most common findings of AIFRS by nasal endoscopy include pale or necrotic mucosa, strong necrotizing nasal crust, and clear or viscous mucus secretions. The most often impacted areas of AIFRS are the middle meatus and middle turbinate, which are followed by the nasal septum and inferior turbinate. Nasal endoscopy showed that necrosis was mostly unilateral, while bilateral necrosis was rare. Few patients had no changes via nasal endoscopy, while most had changes to bone mineral density and sinus opacities via CT [141]. Moreover, nasal endoscopy revealed modifications to the nasal mucosa. According to reports, mucosal edema is an early endoscopic observation that is followed by discoloration, when the pale pink mucosa is replaced with granulation and ulceration. Black discoloration is a sign of late AIFR tissue necrosis, whereas white discoloration is indicative of ischemia brought on by vascular invasion [122].

Histopathological detection: At present, the gold standard for diagnosing AIFRS is tissue biopsy. Traditional formalin-fixed, paraffin-embedded (FFPE) histopathological samples are typically stained with hematoxylin and eosin, periodic acid Schiff (PAS), and Gomori methylamine-silver (GMS) to verify fungal invasion of tissues. Nevertheless, the FFPE method is time-consuming and hinders the rapid diagnosis of AIFRS. For rapid diagnosis of AIFRS, many investigators have argued that frozen section biopsy should be used when evaluating patients who are considered to be at risk [142,143]. The sensitivity of frozen sections for the diagnosis of AIFRS has increased after the addition of modified PAS staining for fungi [144,145]. GMS staining of cryosections is a quick and dependable method for its accuracy, sensitivity, and specificity in diagnosing fungal invasion, with good accuracy when compared to the gold standard FFPE biopsies. Frozen section biopsy is therefore strongly advised for an efficient and quick diagnosis of suspected AIFRS.

##### Treatments of AIFRS

The three common methods for treating AIFRS are reversing the underlying immunodeficiency, antifungal medication, and early surgical debridement. The potential explanations for increased survival after surgery include the availability of culture specimens and earlier tissue diagnosis, penetration of antifungal therapy after resection of necrotic tissue, reduction of fungal burden, and improved postoperative sinus monitoring. Most patients are advised to avoid irritating the necrotic sinus tissue prior to surgery [91]. The surgical strategy of bilateral FESS with debridement of the significantly involved area and antifungal therapy avoids repeat surgery for AIFRS without any change to efficacy [146].

Surgical management: The intranasal technique offers less invasiveness for individuals with poor circumstances and is appropriate for people diagnosed at an early stage of AIFRS. When there is palatal, intracerebral, or intraorbital involvement, open surgery is the better option [147]. However, open surgery did not improve survival, possibly because this patient group had more extrasinus infections. Nonetheless, the survival of patients with orbital or cerebral involvement was less than 6 months, regardless of the clearance of infectious invasive fungi. Intracranial and ethmoid sinus involvement was notably connected to the mortality of AIFRS, possibly due to an increased propensity for fungal infection of the ethmoid sinus, which often spreads directly to the orbit and anterior cranial cavity [148]. Additionally, those who had open surgery might have experienced necrosis and/or a more severe fungal burden. Although investigations have suggested that early endoscopic surgery is key to prognosis, increased time from diagnosis to treatment is an inverse predictor of mortality because patients with a longer onset of symptoms typically have more chronic diseases as compared to those who present with a more aggressive disease course [124]. However, timely diagnosis and treatment are only beneficial for improving morbidity, not mortality [149].

Medical management: Systemic antifungal treatment is also an effective treatment for AIFRS. Amphotericin B and liposomal amphotericin B are currently the main antifungal drugs for AIFRS with *Mucor* infection [141]. In a retrospective study, the survival rate differed among 77.5% and 15.0% of patients with AIFRS who received amphotericin B and liposomal amphotericin B, respectively [116,150]. Furthermore, a systematic review of zygomycosis found that surgery combined with amphotericin B significantly improved survival as compared to either amphotericin B or surgery alone [151]. In addition, an AIFRS patient with acquired immunodeficiency syndrome treated with the newly approved antifungal agent isavuconazole provides a reference for patients with invasive fungal RS either in refractory or intolerant to amphotericin B [152].

Reversal of immunodeficiency: An immune-stimulating therapy (IST) is designed to promote granulocyte, macrophage, and hematopoietic cell maturation. An IST is based on granulocyte colony-stimulating factor (G-CSF), granulocyte-macrophage colony-stimulating factor (GM-CSF), granulocyte infusion, and G-CSF combined with granulocyte infusion. In a systematic review, compared to 44% of untreated patients who died, 88.3% of patients treated with G-CSF survived [151]. Although routinely used for chemotherapy-induced neutropenia, the effectiveness of ISTs for specific opportunistic infections is uncertain. Moreover, an IST was found to significantly improve short-term survival, as determined by multivariate analysis [124]. In addition, it is said that restoring the neutrophil count is necessary to eradicate the infection [153].

#### 4.1.2. CIFRS and GIFRS

The most prevalent invasive fungal RS is AIFRS, which is classified into two types: CIFRS and GIFRS. CIFRS is a relatively rare invasive fungal disease involving the paranasal sinuses and nasal cavity with an uncertain pathogenesis. Histologically, sparse inflammation, sporadic vascular infiltration, and extensive hyphae formation are the main features of CIFR [111]. As compared to AIFRS, CIFRS progresses slowly over a period of months to years [154]. A retrospective analysis found that compromised immunity, diabetes mellitus, and extended corticosteroid use are significant risk elements for CIFRS [155]. In another investigation, one or more predisposing factors were found in 43.1% of CIFRS patients [156]. Unlike AFIRS, the reversal of underlying immunodeficiency is not necessary for CIFRS, which also occurs in immunocompetent individuals. Clinical symptoms of CIFRS are nonspecific and may include facial pain, pressure, runny nose, nasal congestion, epistaxis, and symptoms related to the invasion of the orbit or brain [157]. *A. fumigatus*, *A. flavus*, *Mucor*, and *C. albicans* are the frequent species causing fungal infection [158]. In addition, CIFRS caused by the rare fungi *Paecilomyces variotii* has also been reported [159]. CIFRS is histopathology characterized by the fungal invasion of sinus tissue, positive fungal culture, soft tissue infiltration, and irregular bone destruction detected via CT or MRI. The thickening of the sinus mucosa and sinus opacification are the most frequent CT findings [160].

Unlike CIFRS, granulomatous inflammation along with severe fibrosis is a characteristic of GIFRS. Large numbers of multinucleated giant cells and fewer numbers of epithelioid, lymphocyte, plasma, eosinophil, and neutrophil cells formed these noncaseating granulomas, while the presence of fungal hyphae is rare. According to reports, GIFRS is prevalent in hot, arid nations like Sudan, Pakistan, and India. *A. flavus* has been isolated from most granulomatous fungal RS [161].

Between CIFRS and GIFRS, there are no appreciable differences in survival rates, clinical profiles, or demographic characteristics. However, in the granulomatous group, the median time between the onset of symptoms and diagnosis was 4 months, a substantially greater duration than that in the chronic group [156]. Diagnosis and treatment are similar to AIFRS. Surgery and antifungal drugs are commonly used for the treatment of CIFRS and GIFRS. Similar to AFIRS, interventions include open surgery, endoscopy, and combined surgery with the use of antifungals [115].

### 4.2. Non-Invasive Fungal RS

#### 4.2.1. AFRS

AFRS is a subtype of CRSwNP that is characterized by specific antifungal IgE production, eosinophil-rich mucus, and characteristic CT and MRI findings of the paranasal sinuses [162]. AFRS usually occurs in immunocompetent atopic persons, strikes more frequently in regions with warm temperatures and high humidity, impacts younger people (ages 21–33), and affects men more than women [163]. The most prevalent pathogenic fungi of AFRS are the *Aspergillus* species and dematiaceous fungi, including *Alternaria*, *Bipolaris*, *Cladosporium*, and *Curvularia* [162,164,165].

##### Pathogenesis and Host Immune Response

AFRS involves molecular signaling in antifungal immunity, mucosal barrier physiology, and fungal activation, which result in an excessive type 2 immune response featured by the production of the cytokines IL-4, IL-5, and IL-13, and upregulation of adaptive immune responses characterized by elevated IgE levels [166,167]. Previous investigations suggest that the characteristics of AFRS are pathogenic fungi, elevated IgE levels, and systemic allergy to fungal antigens [168,169,170]. However, recent investigations confirmed a high positive fungal culture rate for people with and without AFRS [171,172]. For both AFRS and CRS patients without a fungal allergy, there was no significant distinction in the levels of fungal-specific IgE or in the generation of fungal-specific peripheral lymphocytes. In addition, reactivity to fungal antigens and the presence of fungi-specific IgE and IgG can occur in both non-allergic and allergic fungal RS [173,174,175]. Immunohistochemical analysis of tissues from AFRS individuals and non-allergic eosinophilic fungal RS showed no variations in the numbers of mast cells and eosinophils [176].

Epithelial activation with concomitant EC apoptosis and loss of EB integrity: The EB of the nasal mucosa is composed of a group of cells, peptides, and proteins that are intricately connected to the immune response [163]. Alterations to EB function are considered important for the pathogenesis of AFRS. Decreased transepithelial electrical resistance has been observed in cultured sinus ECs from patients with AFRS. Moreover, the decreased expression of the TJ proteins occludin and junctional adhesion molecule-A and the increased expression of the leaky TJ protein claudin-2 were linked to increased epithelial permeability [177]. In addition, fungal proteases can cause structural changes to epithelial junctions and increase epithelial permeability [178,179]. In addition, the protein and peptide components of the natural barrier of the nasal mucosa, including defensins, antimicrobials, lysozyme, lactoferrin, and SPLUNC1, are also involved in the defense against pathogens and mucosal injury [180,181]. The expression levels of lactoferrin, surfactant protein, and antibacterial S100 protein, histatin-1, and histatin-3 are reduced in AFRS patients [182,183,184,185]. Therefore, immunodeficiency of antimicrobial peptides in AFRS patients results in increased susceptibility of the EB to fungi and decreased clearance of proteases and mycotoxins [181].

Release of proinflammatory chemokines and cytokines: The respiratory epithelium of the sinuses and lower airways releases innate inflammatory cytokines (also called epithelial-derived cytokines), including thymic stromal lymphopoietin (TSLP), IL-33, and IL-25, which contribute to type 2 inflammation. TSLP induces the production of IL-5 and IL-13 by type 2 cells and type 2 intrinsic lymphoid cells (ILC2s). The ST2 receptor of IL-33 exists on eosinophils, T cells, innate lymphoid cells, and mast cells. IL-33, through the ST2 receptor, can promote Th2 polarization, eosinophil production, and type 2 inflammation [186]. IL-25, a distinct member of the IL-17 cytokine family, is also referred to as IL-17E [187]. Chemosensory cells (SCCs) are the main source of IL-25 in the human sinus epithelium [188]. As compared to normal controls, expression levels of SCCs and ILC2s are increased in CRS patients [189]. An elevated level of the IL-33 receptor in AFRS patients has been connected with the increased gene expression of eosinophils and mast cells [167].

Animal and cellular models have confirmed that epithelial-derived cytokines promote type 2 inflammatory responses. In asthma, TSLP and IL-33 cooperatively promote ILC2 activation and induce congenital allergic inflammation [190]. Airway ECs release IL-33, IL-25, and TSLP in a mouse model of allergic airway inflammation triggered by fungi. ILC2s then quickly produce type 2 cytokines, which cause airway eosinophilia, mucus formation, and hyperresponsiveness [191]. In a model, ILC2 was shown to significantly reduce TJ integrity via the expression of IL-13. In addition, epithelial electrical resistance was decreased and isothiocyane-dextran fluorescein permeability was increased, consistent with the decreased mRNA and protein expression of TJ proteins [192,193].

Another crucial aspect of type 2 inflammation is eosinophil activation. Mast cells and eosinophils are recruited and activated by IL-9 and IL-5, respectively. When IL-4 and IL-13 are present, B cells switch their class and start producing IgE. The specific IgE antibodies attach to basophil and mast cell surface high-affinity receptors [194]. In addition, eosinophils can release superoxide anions, hydrogen peroxide, and eosinophil peroxidase, which damage the ECs of the nasal mucosa [195]. The ratio of dendritic cells is increased in CRS individuals [196]. The production of CCL20 and CCL2 by nasal ECs in response to stimulation by other antigens, such as fungi, recruits immature dendritic cells from the peripheral blood. After stimulation with epithelial-produced TSLP, dendritic cells generate large amounts of CCL17, CCL18, and CCL22, which are involved in the Th2 cell recruitment process [197]. Mast cells are crucial initiators of inflammatory responses. After receiving allergen signals, mast cells promote vascular dilation and permeability by secreting pro-inflammatory chemokines and other molecules that promote the recruitment and trafficking of immune cells to the sites of infection and inflammation. These immune cells interact with each other to maintain and prolong inflammation [195,198,199].

**Figure 3:**
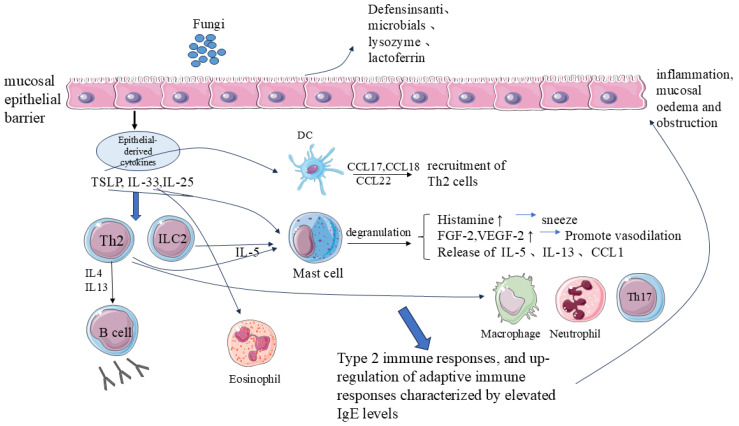
The immune response in AFRS. Fungal colonization in the nasal cavity and paranasal sinuses leads to epithelial cell activation followed by epithelial cell apoptosis and the loss of epithelial barrier integrity. Various proteins and peptides are released to defend against pathogens and mucosal damage, in addition to the increased production of epithelial-derived cytokines, including TSLP, IL-25, and IL-33. The subsequent inflammatory cascade leads to eosinophilia, formation of nasal polyps, and mucus production. Tissue edema and mucus production caused by the infiltration of various immune cells and increased secretion of a series of inflammatory mediators damage the epithelial barrier, leading to impaired fungal clearance and increased risk of invasion, which finally manifests as AFRS. Note: TSLP: thymic stromal lymphopoietin; IL33: interleukin 33; IL-25: interleukin 25; ILCs: type 2 intrinsic lymphoid cells; Th2: T helper 2 cell; Th17: T helper cell 17; IL-4: interleukin 4; IL-13: interleukin 13; IL-5: interleukin 5; DC: dendritic cell; FGF-2: fibroblast growth factor 2; VEGF-2: vascular endothelial growth factor receptor 2; CCL1: C-C Motif Chemokine 1. ↑: increased secretion or expression.

Exposure to conditions that allow fungal spores to enter the sinuses, where the spores can germinate into immunogenic fungal hyphae and cause EB dysfunction as well as the release of TSLP, IL-25, and IL-33, epithelial-derived cytokines, is what causes AFRS. As a result, the type 2 immune response becomes compensatively overstimulated, triggering an inflammatory cascade that produces mucus, eosinophilia, and nasal polyps. The infiltration of various immune cells and the increased secretion of inflammatory mediators cause tissue edema and mucus production, which disrupt the EB and increase the risk of fungal invasion. This vicious cycle eventually progresses to AFRS [200,201,202]. There is a significant correlation between the obvious type 2 inflammation observed in AFRS and the disruption of innate immune signaling and EB function (Figure 3).

##### Diagnosis

Nasal polyps almost invariably accompany AFRS, which manifests as CRS symptoms unresponsive to traditional medical treatment. However, as compared to CRSwNP, the nasal discharge of AFRS patients is usually as thick as peanut butter with a greenish-brown mucoid appearance (Table 1).

The complications that may occur in AFRS patients include visual disturbances, exophthalmos, facial deformities, and intracranial sequelae, such as stress-induced intracranial neuropathy and intracranial abscesses. Sinus CT images of individuals with AFRS usually display nearly complete opacity with heterogeneous radiation density of the sinus soft tissues. CT findings include sinus dilatation, filling with high-density material, and erosion of the sinus wall bone. Patients with AFRS can have a 20% to 90% rate of bone degradation [209]. The initial Bent–Kuhn diagnostic criteria include (1) nasal polyps, (2) stained fungi, (3) eosinophilic mucin, with no fungal invasion of paranasal sinus tissue, (4) type I fungal allergy, and (5) characteristic radiological findings of poor soft tissue density on CT images. Type I hypersensitivity and characteristic CT findings are the only unique factors of the Bentt–Kuhn criteria [210].

##### Treatment

Surgery is the mainstay of AFRS treatment, which not only can re-establish ventilation and eliminate antigenic stimulation in patients with AFRS but also provide broader access to monitoring, clinical debridement, and the application of topical drugs. Local and systemic corticosteroids are the foundation of AFRS drug treatment. Oral corticosteroids can enhance olfaction and endoscopic scores, reduce polyp formation, and decrease blood eosinophil and IgE level. However, extended use of oral corticosteroids can cause significant side effects. Fortunately, oral corticosteroids cause relatively few adverse events in patients with AFRS [211]. In contrast to oral steroids, topical nasal steroid sprays have a favorable safety profile and can achieve effective drug concentrations in the sinus mucosa without adverse consequences. In addition, topical steroid delivery through novel steroid-eluting sinus implants is also a safe option. Clinical investigations have shown that the use of different steroid-eluting sinus implants can enhance postoperative outcomes of FESS and prevent the recurrence of nasal polyps after sinus surgery [212,213]. An evidence-based review strongly recommends the use of standard topical nasal steroids and nonstandard topical nasal steroid therapy for the treatment of CRS [214]. In addition to standard medical treatment with steroids (systemic/topical), antifungal agents have been applied to decrease the recurrence rate by preventing postoperative fungal development. Several randomized controlled clinical trials of topical/systemic steroids and itraconazole for the treatment of AFRS have recommended itraconazole as a successful substitute to steroids for the postoperative treatment of AFRS [215,216]. In addition, the efficacy of preoperative itraconazole treatment is significantly better than that of postoperative itraconazole, although the recurrence rate before and after surgery was similar [217]. A study to evaluate the efficacy of systemic and local antifungal drugs (alone and in combination) to prevent the recurrence of AFRS after FESS found that local use of fluconazole as a nasal spray and the combination of oral itraconazole and fluconazole considerably reduced the recurrence of AFRS after FESS [218]. Following the treatment of AFRS with antifungal drugs, there was no differences in quality of life, efficacy outcomes, CT scan score, and endoscopy score with the placebo group [219]. Common side effects of antifungal drugs include elevated liver enzymes, congestive heart failure, nausea, rash, headache, malaise, fatigue, and edema. Hence, the use of antifungal drugs is dependent on the overall condition of the patient. Nonetheless, oral antifungal agents are a feasible option for treatment of refractory AFRS, although efficacy, safety, and dosage must be further investigated.

Because AFRS is essentially a type I hypersensitivity reaction, immunotherapy may attenuate the immune response to fungi and reduce the disease burden. Allergen immunotherapy was shown to notably enhance endoscopic mucosal staging, quality of life, and dependence on systemic and oral corticosteroids. Moreover, low-dose subcutaneous fungal immunotherapy targeting fungal allergens could be efficient for the treatment of AFRS. In addition, allergen immunotherapy caused no serious short- or long-term adverse effects in these patients [170,220,221,222,223]. Adverse effects and complications did not differ between patients and healthy groups, and fungal immunotherapy was associated with fewer serious adverse effects [224,225]. As part of the treatment plan, allergen immunotherapy that targets fungi should be taken into consideration as it is anticipated to decrease the recurrence of AFRS. Based on this evidence, allergen immunotherapy may be beneficial for patients with AFRS. However, immunotherapy combined with other medical therapies did not reflect the real impact of immunotherapy on AFRS patients. Therefore, more prospective research is needed to decide the effectiveness of allergen immunotherapy for the treatment of AFRS [226].

#### 4.2.2. Saprophytic Fungal Infestation and Fungal Ball

As compared to AFRS, the incidence is lower for saprophytic fungal infestation and fungal ball. Saprophytic fungal RS has been described as the fungal colonization of nasal secretions or crusted mucosa, which is caused by inhalation exposure of the inflamed or crusted nasal mucosa with fungal spores after surgical intervention. Restricted to superficial crusts/mucosa within the nasal cavity, fungal hyphae does not enter the tissue through epithelial invasion [115,227]. Although there is no typical clinical disease course, there is usually an unpleasant nasal odor. This type of fungal RS is considered to develop into the fungal ball type [228].

Described as a dense collection of fungal hyphae outside the mucosa, a fungal ball histologically exhibits fungal colonization but no invasion and generates minimal mucosal irritation or response [229]. Fungal balls develop slowly and have no specific symptoms. A fungal ball usually involves only a single sinus in the great majority of cases. The maxillary and ethmoid sinuses and the sphenoid sinus are the most often involved sites [230]. The symptoms change based on which sinuses are affected. Common clinical manifestation of maxillary sinus fungal ball includes facial pain, rhinorrhea, nasal obstruction, and nasal odor. Headache and nasal obstruction are common clinical manifestations of ethmoid fungal ball. Sphenoid sinus fungal ball infections are characterized by recurrent retro-orbital pain with visual disturbances [229]. Fungal ball in the sinus occurs mainly in the elderly population, especially women, although the incidence and recurrence rates are low [231]. In addition, invasive fungal RS can develop from non-invasive fungal balls, particularly in older and immunocompromised people [232]. Retrospective analysis shows that most patients are immunocompetent, and about half have dental pulp treatment [198,200]. The pathogenesis of fungal ball in the paranasal sinuses remains unclear. There are two hypotheses about the origin of fungal ball, including sinonasal anatomical variations and dental factors [227]. Anatomical obstruction of the sinus ostium is one example of an anaerobic environment that can promote the growth of fungi [233]. The high frequency of concha bullosa in the fungal ball group of the maxillary sinus may indicate a pathogenic role for this anatomical defect. Because Concha bullosa alters nasal airflow, spore deposition into the maxillary sinus may be facilitated [234]. Some investigations also found a significant difference in the rate of endodontic therapy between patients with fungal ball and those without it [235]. The common species connected with fungal ball include *A. fumigatus*, *A. flavus*, and occasionally *Chrysosporium* and *Penicillium* [198]. The clinical pathological diagnostic criteria for fungal ball in paranasal sinuses are (1) imaging evidence of sinus opacification with or without calcification, (2) a mucoid or clay-like substance with purulence, (3) hyphomycetes (dense aggregates of hyphae separated from the sinus mucosa), (4) non-specific chronic mucosal inflammation with no eosinophil dominance, granulomas, or allergic mucin, and (5) no histological or microscopic evidence of fungal invasion of the mucosa, blood vessels, or bone [236]. Primary treatment of fungal ball includes the removal of fungal components, systemic use of antifungal drugs, and intraoperative and postoperative flushing of the nasal cavity with antibiotic saline.

## 5. Conclusions

Infectious RS is caused by pathogenic microorganisms and can be triggered by imbalances among the fungi, respiratory viruses, and bacterial species of the microbiome of sinuses (Table 2). With continuous attention to the microbiome in the sinus cavity, microbiota imbalance has been widely considered to be one of the mechanisms of CRS [237]. In addition, mixed infection of multiple pathogenic microorganisms is common in RS [238,239,240]. Damage to the host immune system or local microecology caused by unbalanced growth of one pathogenic microorganism renders the host more susceptible to infection by other pathogenic microorganisms. Therefore, it is necessary to focus on the occurrence of infectious RS after infection with other pathogenic microorganism, early diagnosis, and early treatment.

Pathogens disrupt the EB of the nasal mucosa and activate the immune response, which is considered an important mechanism in the pathogenesis of RS. These immune responses not only recruit neutrophils and macrophages to activate the innate immune response but also stimulate the release of IL-13, IL-5, IL-4, and IL-9, thereby promoting type 2 inflammation. The infiltration of various immune cells and the increased production of an array of inflammatory mediators ultimately leads to tissue edema and mucus generation. Meanwhile, damage to the EB increases the risk of pathogen invasion. In addition, recent investigations have confirmed the increased production of IL-17A in CRS patients. The overexpression of IL-17A promotes CRS through tissue remodeling, eosinophilic accumulation, and neutrophilic infiltration. Although significant progress has been made to elucidate the pathogenesis of infectious RS, unique biomarkers and evidence-based treatment options are still lacking for infectious RS; thus, further investigations are warranted.

## Figures and Tables

**Table 1:** Differences between AFRS and CRSwNP.

	AFRS	CRSwNP	Reference
Secretions	Thick, peanut butter consistency	Thick	[1]
Serum IgE	High	Lower than AFRS	[203]
CT imaging	Opacification with hyperdense areas and central hyperattenuation due to allergic mucin	Opacification	[204,205]
Fungi	*Aspergillus* species and melanin-producing fungi	*Alternaria* and *Cladosporium*	[162,206,207]
HLA	HLA-DQB1*0301 and HLA-DQB1*0302 were highly correlated	HLA-DQB1*0301 and HLA-DQB1*0302 less prominent	[208]

Note: *: In HLA nomenclature, use an asterisk * to separate the gene from the 4 numeric regions.

**Table 2:** Comparisons of RS caused by viruses, bacteria, and fungi.

	Clinical Characteristics	Common Types of Pathogens	Pathogenesis and Host Immune Response	Diagnosis	Treatment	Reference
Viral RS	Mostly acute and usually lasts less than 10 days. Common cold symptoms, a blocked nose or a feeling of congestion, nasal discharge, green/yellow colored mucus or even pus, or facial pain or pressure, headache, and reduction/loss of smell.	Coronavirus, adenovirus, human rhinovirus, influenza virus, and respiratory syncytial virus.	Binding to the sinonasal epithelium through specific receptors, then entering cells and replicating. Triggering intrinsic immune response dominated by IFN and type 2 inflammatory response.	The clinical manifestations of sinus inflammation, sinus CT, and molecular detection technology.	Antiviral drug treatment and surgery.	[1,8,9,11,12,13,14,35,36,38,39,40,41,52,53,54,55,57,58,60,61,62,63,64,65,66,67,68,69,70,71]
Bacterial RS	Patients feel more unwell than a typical cold and may result in severe facial pain located around one side, a high fever (>38°), mucus that looks green, yellow, or rusty brown. Severe patients usually present with symptoms such as nasal blockage/congestion, nasal discharge, altered sense of smell, and facial pain.	*S. aureus*, *S. pneumoniae*, *M. catarrhalis*, *P. aeruginosa*	Bacterial components and virulence factors. Regulates innate and adaptive immunity by disrupting tissue barrier function, promoting impaired mucociliary clearance, driving polyps, and promoting type 2 inflammation.	Microbial culture, patient’s clinical symptoms, and sinus CT.	Steroids, antibiotics, nasal saline irrigation, surgery, immunoglobulin replacement.	[1,2,72,73,74,75,76,77,78,79,80,81,82,83,84,85,86,87,88,89,90,91,92,93,94,95,96,97,98,99,100,101,102,103,104,105,106,107]
Fungal RS	The most common clinical manifestations were ocular swelling, exophthalmos, and headache.	Typical *Mucor*, *Aspergillus*, and atypical fungi (*Alternaria*, *Candida*, *Fusarium*, *Paecilomyces*, *Scedosporium* and *Scopularopsis*.)	AFRS: epithelial activation with concomitant EC apoptosis and loss of EB integrity, an excessive type 2 immune response characterized by cytokines, such as IL-4, IL-5, and IL-13, and an upregulation of adaptive immune responses characterized by elevated IgE levels.	In addition to the above mentioned, fungal RS can be diagnosed through special staining techniques such as periodic acid Schiff staining.	Early surgical debridement, antifungal therapy, and reversal of underlying immunodeficiency.	[109,121,122,123,137,138,139,140,141,142,143,144,146,173,182,183,186,187,189,193,194,195,196,197,198,199,200,201,202,203,204,205,206,207,219,220,221,222,223,224,225,226,227,228,229,230,231,232,233,234,235]
